# Chromosome-1 abnormalities in Childhood B-Lymphoblastic Leukemia – An analysis with reference to clinical variables and survival outcome

**DOI:** 10.12669/pjms.40.2(ICON).8946

**Published:** 2024-01

**Authors:** Neelum Mansoor, Naila Rafiq, Saba Jamal, Aamir Ehsan

**Affiliations:** 1 Neelum Mansoor, FCPS. Section Head Cytogenetics, Indus Hospital and Health Network, Karachi, Pakistan; 2 Naila Rafiq, FCPS. Consultant Pediatric Hematology-Oncology, Indus Hospital and Health Network, Karachi, Pakistan; 3 Saba Jamal, Diplomate American Board of Anatomic and Clinical Pathology, Diplomate American Board of Hematology, Senior Director Pathology and Blood Transfusion Services, Indus Hospital and Health Network, Karachi, Pakistan; 4 Aamir Ehsan, MD. Medical Director, CorePath Laboratories, San Antonio, Texas, USA

**Keywords:** Chromosome 1, Leukemia, Cytogenetics, Chromosomal aberrations, Survival outcome

## Abstract

**Background:**

Chromosome-1 abnormalities (C1As) are common genetic aberrations in hematological malignancies. We sought to evaluate significance of these abnormalities with reference to clinical characteristics and survival outcome in a pediatric B-Lymphoblastic Leukemia (B-ALL) cohort.

**Methods:**

This is a retrospective study conducted in cytogenetic section of Indus Hospital and Health Network. Data was retrieved from October 2020 to July 2022 for childhood B-ALL cases exhibiting C1As. Chromosome analysis was performed on Cytovision MB8 using G-banded metaphases derived from unstimulated bone marrow culture. Results were recorded according to the International System for Human Cytogenetic Nomenclature (ISCN-2020). Data analyzed using SPSS, version 24.0.

**Results:**

C1As were observed in 60/450 (13.3%) cases of B-ALL. Among C1As, 29 (48%) cases had t(1;19). There were 13 (45%) balanced and 16 (55%) unbalanced translocations. The aberrations without t(1;19) were seen in 31 (52%) cases including 1q duplication with hyperdiploidy in 14 (45%) cases. The median age for C1As with and without t(1;19) was eight years and six years while the median leukocyte count was 32 x 10^9^/L vs. 17 x 10^9^/L. Event-free survival (EFS) for cases with and without t(1;19) was 69% and 74.2% respectively.

**Conclusion:**

Despite the fact that the t(1;19) positive group had a higher median age, a higher white cell count and more CNS positives, the difference in EFS is statistically insignificant when compared to the t(1;19) negative cases. Furthermore, we found a survival difference between balanced and unbalanced t(1;19) groups, which is statistically insignificant but warrants large-scale prospective studies for further understanding.

## INTRODUCTION

The most common childhood cancer is acute lymphoblastic leukemia (ALL), with 80-85% of cases coming from the B lineage.^1^,^2^ Childhood B lymphoblastic leukemia (B-ALL) usually has a good prognosis, with a cure rate approaching 90% in high-income countries.^3^,^4^ This improvement in outcome is a result of appropriate risk stratification, the introduction of risk-adjusted treatment protocols, and better supportive care. In addition to the National Cancer Institute (NCI) based risk factors of age and white cell count, disease immunophenotype (B-cell versus T-cell), central nervous system (CNS) involvement, treatment response, and cytogenetic aberrations are the key determinants of disease outcome.^4^-^6^

The study of chromosomal abnormalities is one of the essential components in understanding the genetic basis of ALL. A number of recurring chromosome abnormalities define genetic subtypes of B-ALL that are associated with specific clinical outcome. The abnormalities of chromosome-1, which is the largest human chromosome, are commonly reported in hematological malignancies such as multiple myeloma and myeloid malignancies. A variety of structural and numerical chromosome-1 aberrations (C1As) are described in these hematological disorders with reference to clinical features and prognosis.^7^,^8^ In childhood B-ALL, translocation t(1;19)(q23;p13) causing *TCF3-PBX1* fusion gene is one of the well-known C1As. Historically, this abnormality was considered as a poor prognostic factor, but recent studies show an improved outcome with intensive chemotherapy protocols.^9^ With the exception of the knowledge of t(1;19) translocation, there is a paucity of information regarding various structural and numerical chromosome-1 abnormalities in childhood ALL. As C1As have been linked to disease progression in myeloproliferative neoplasia and have a poor prognostic impact in multiple myeloma, it is crucial to look for various C1As in childhood B-ALL to see if there is a relationship between clinical factors and disease outcome.

In the current study, cases of childhood B-ALL with different C1As were analyzed. The chromosomal abnormalities and clinical features of these cases were correlated and the impact of these abnormalities on patient prognosis was discussed.

## METHODS

This is a retrospective, observational and non-therapeutic study conducted in the cytogenetic section of Clinical laboratories of Indus Hospital and Health Network (IHHN). IHHN is a tertiary care nonprofit, nongovernmental organization having one of the largest pediatric oncology units in the country dealing with more than 1200 new patients of childhood cancer per year.The patients’ Medical Record number was used as their personal-identifying information. Data of all patients reviewed from one to sixteen years of age who were diagnosed with B-ALL. Infantile leukemia cases (age < 1 year) were excluded from the analysis. The diagnosis was confirmed by immunophenotyping using flow cytometric analysis of bone marrow aspirate or blood samples or immunohistochemistry on bone marrow biopsy.

### Ethical Approval

It was obtained from hospital IRB Ref: (IRB_IRB_2021_06_012) and then, the hospital’s electronic medical record was reviewed from October 2020 to July 2022.

### Inclusion & Exclusion Criteria

Among 473 diagnosed cases of B-ALL, 450 were found to be eligible for study analysis. We excluded 23 cases from the study due to the absence of cytogenetic sample with initial diagnostic workup (six cases) or due to failure of marrow culture for cytogenetic studies (17 cases). For chromosomal analysis, bone marrow aspirate samples collected in sodium heparin were used and unstimulated cultures for 24 hours were setup. After culturing, metaphases were arrested by colcemid and then treated with hypotonic solution. This was followed by fixation using preformed fixatives (Methanol: Glacial acetic acid). After fixation slides were made and GTG banding was performed. By using Cytovision MB8 software (Leica Biosystems, Germany), total 20 GTG banded metaphase cells were analyzed. Results were recorded according to the International System for Human Cytogenetic Nomenclature (ISCN-2020).

### Statistical Analysis

Data was analyzed by using SPSS, version 24.0 (IBM Corp., Armonk, NY). Descriptive statistics like median, and interquartile range (IQR), were computed for age and white cell count, as appropriate. Frequency and percentage were calculated for qualitative variables such as sex, central nervous system status, prednisolone response, various cytogenetic aberrations and disease outcome.

The statistical tests of Kruskal-Wallis H, and Fisher-exact were used, as needed, to measure the association between various categories of C1As and clinical parameters including age, WCC, CNS status, etc. A p-value <0.05 was considered statistically significant.

Events evaluated as endpoints were complete remission (CR) and event-free survival (EFS). CR was defined as <5% blasts in the bone marrow by the end of induction. EFS was defined as time elapsed from date of diagnosis to occurrence of an event including relapse, death due to any cause, and abandonment. The Kaplan-Meier method was used to estimate the survival probability, and log-rank test was used to determine statistical significance.

## RESULTS

**Fig.1:**
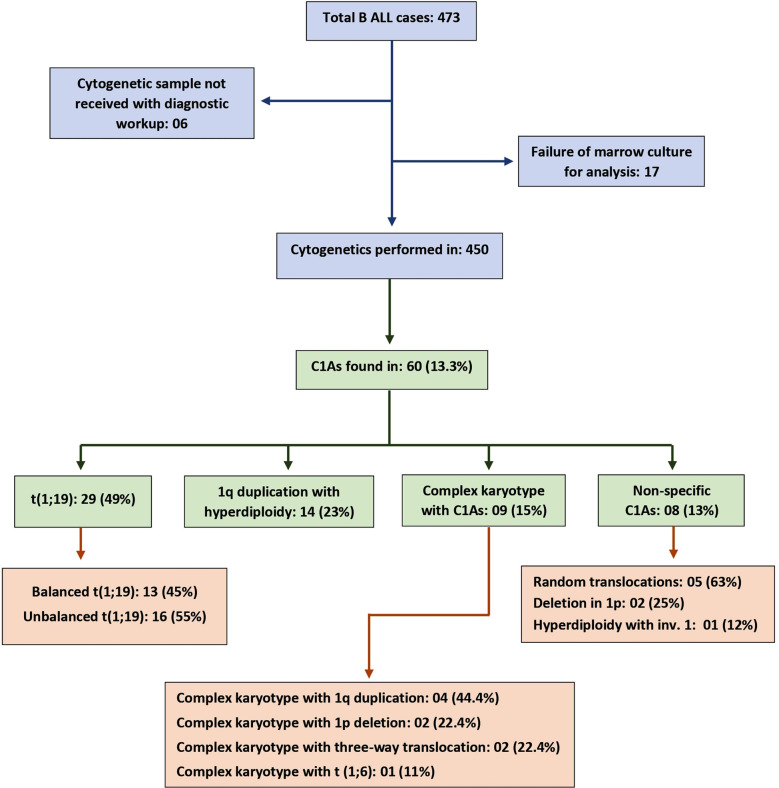
Distribution of chromosome-1 abnormalities.

Out of 450 cytogenetically evaluated patients, 60 (13.3%) showed chromosome-1 abnormalities. Among those who tested positive for C1As, 52% were male and 48% were female. The median age of the cohort was seven years, and the median hemoglobin (Hb), WCC, and platelet counts were 7.25 g/dl, 23.15 x 10^9^/L, and 20 x 10^9^/L, respectively. Depending on the type of aberrations, C1As were broadly stratified into four categories including cases with t(1;19), 1q duplication with hyperdiploidy, complex karyotype with C1As, and non-specific C1As. The occurrences and further delineation of these categories are shown in Fig.1. These aberrations were analyzed with respect to the patient’s demographics and different clinical variables as shown in Table-I. Although statistically insignificant, an interesting finding was the higher incidence of unbalanced t(1;19) in female patients (62.5% versus 37.5% cases). Likewise, the category of unbalance t(1;19) had highest median WCC (43 x 10^9^/L), the highest CNS positive cases (18.8%), and a large proportion of NCI HR patients. The median age differed amongst the categories of C1As, with non-specific chromosomal abnormalities having the lowest median age of 4.5 years (p-value 0.061). Similarly, there was a variance in hemoglobin, white cell counts and platelet counts across categories as shown in Table-I. However, these findings were not statistically significant (p-value: >0.05). Data of different clinical prognostic variables was evaluated. In a total cohort, 11.6% (7/60) cases were CNS positive, 15% (9/60) were prednisone poor responder and 55% (33/60) were NCI high risk cases. The association between these prognostic variables and different categories of C1As was not statistically significant. There were two abandonments and two deaths during the induction phase of chemotherapy, therefore post-induction bone marrow assessment was applicable in 56 cases and all achieved CR.

**Table-1:** Characteristics of patients with reference to various C1As.

Characteristics	Cases with t(1;19)		Complex karyotype with C1As	Duplication of 1q with hyperdiploidy	Non-specific C1As	P-value

	Balanced translocation	Unbalanced translocation				
No. of patients	13	16	09	14	08	-
***Sex (n) (%)***						0.710^a^
Male	08 (61.5)	06 (37.5)	05 (55.6)	07 (50)	05 (62.5)	
Female	05 (38.5)	10 (62.5)	04 (44.4)	07 (50)	03 (37.5)	
***Age (years)***						0.053^b^
Median	08	09	08	5.5	4.5	
(IQR)	(03-11)	(07-13.5)	(06-09)	(03-07)	(3.25-10.75)	
***Hemoglobin (gm/dl)***						0.411^b^
Median	08	7.0	9.4	6.5	7.8	
IQR	(6.5-10)	(6.4-7.9)	(4.2-10.4)	(3.2-8.3)	(6.9-9.4)	
***WCC (x10^9^/L)***	26.5					0.272^b^
Median	(11.1-78.3)	43.0	18	17.8	28.1	
IQR		(13.2-104.3)	(7.4-56.8)	(8.4-31.7)	(4.0-76.5)	
***Platelets (x10^9^/L)***						0.703^b^
Median	17	19.5	35	37.5	17	
IQR	(07-62.5)	(14-58.5)	(8.5-126.5)	(9.7-53.5)	(06-56.25)	
***CNS Status (n) (%)***						0.855^a^
Negative	12 (92.3)	13 (81.3)	08 (88.9)	12 (85.7)	08 (100)	
Positive	01 (7.7)	03 (18.8)	01 (11.1)	02 (14.3)	00	
***Prednisone response (n) (%)***						0.561^a^
Good	11 (84.6)	14 (87.5)	08 (88.9)	10 (71.4)	08 (100)	
Poor	02 (15.4)	02 (12.5)	01 (11.1)	04 (28.6)	00	
***NCI Risk (n) (%)***						0.313^a^
Standard Risk	06 (46.2)	04 (25)	04 (44.4)	09 (64.3)	04 (50)	
High Risk	07 (53.8)	12 (75)	05 (55.6)	05 (35.7)	04 (50)	

a Fischer Exact test,

b Kruskal-Wallis H test, IQR: Interquartile range, WCC: White cell count, CNS: Central nervous system, NCI: National cancer institute.

**Fig.2:**
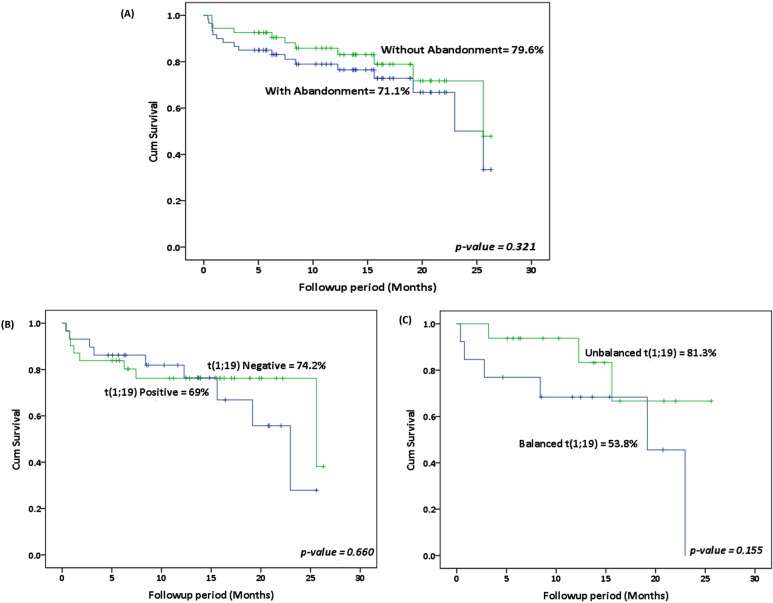
Event free survival (EFS). (A) Total cohort with and without abandonment. (B) Cases with and without t(1;19) including abandonment. (C) Cases with balanced versus unbalanced t(1;19) including abandonment.

With the median follow up of 12.6 months (IQR 5.8–17.2 months), the Kaplan-Meier estimate of EFS with and without abandonment for the entire cohort at five years was 71.1% and 79.6% respectively. The survival outcome was also analyzed with reference to different categories of C1As. EFS for cases with and without t(1;19) was 69% and 74.2% respectively. Moreover, balanced and unbalanced translocations were used to further categorize cases of t(1;19), and EFS was found to be 53.8% and 81.3%, respectively. These results are shown in Fig.2.

## DISCUSSION

Cytogenetic abnormalities such as chromosomal structural rearrangements and aneuploidy are the most common findings in childhood ALL and are observed in approximately 70-80% of cases.^10^ For a risk-adapted therapy and the improvement of outcomes, the studies on their prognostic value are essential. We analyzed the clinical significance of C1As in a cohort of childhood B- ALL which is the most prevalent cancer in this age group.

In our retrospective analysis, C1As were observed in 13.3% (60/450) cases of B-ALL. Although overall incidence of C1As have been commonly reported in many adult-onset hematological malignancies, including chronic myeloproliferative disorders (up to 28%) and multiple myeloma (45%) but there is a paucity of data for childhood ALL.^7^,^11^ However, there are studies on specific chromosome-1 abnormalities such as t(1;19) and 1q duplication in childhood B-ALL. The two most frequently observed C1As in our cohort were t(1;19) and duplication 1q with hyperdiploidy, with prevalence of 49% (29/60) and 23% (14/60), respectively. The overall occurrence in total cohort was 6.4% (29/450) for t(1;19) aberration and 3.1% (14/450) for duplication 1q with hyperdiploidy, both of which were found to be comparable to previously published data.^12^,^13^ The categories of C1As were analyzed with reference to different clinical and prognostic factors.

Our results showed a median age of eight years for balanced t(1;19) cases and nine years for unbalanced t(1;19) cases. These findings contrast with Berlin–Frankfurt–Munster (BFM)-based studies, in which the median ages for balanced and unbalanced t(1;19) cases were six years and four years, respectively.^12^ In contrast to previous results, we observed lower median values for hemoglobin and platelets, and higher median values for WCC in both the categories of balanced and unbalanced t(1;19).^12^,^14^ Furthermore, we had 65.5% NCI HR cases in the t(1;19) group, 36% in duplication 1q category and 55% in the entire cohort. These figures are much higher than the numbers reported by previous studies.^12^,^13^,^15^ These differences can partly be explained by a delayed recognition and diagnosis of patients in our resource-limited settings, however further studies may be required to determine the other biological differences.

The category of 1q duplication with hyperdiploidy has 14% (2/14) CNS positive cases in our cohort. This finding contrasts with an Egyptian investigation that found no CNS involvement in a subset of 14 patients with 1q duplication.^13^ With respect to the categories with positive and negative t(1;19), CNS positivity was observed in 13.7% and 9.6%, respectively. The influence of t(1;19) on CNS involvement yields contradictory results. Data from the Nordic countries and a study from China demonstrate no correlation between CNS positivity and t(1;19); however, an investigation from Argentina showed a higher incidence of CNS involvement in t(1;19) patients.^12^,^14^,^16^ Although literature also reports an increased risk of CNS relapse with t(1;19), no CNS relapse has been observed in our cohort so far.^17^ The short follow-up time, on the other hand, may be a limiting factor in our cohort’s meaningful interpretation of CNS relapse data.

In terms of treatment response, we found no statistically significant variations in prednisone response or CR rates amongst the various categories of C1As. This data is in concordance with other studies. 14, 18 Without abandonment, EFS in our cohort is 79.6%, which is poor in contrast to regional data from China and data from developed countries.^14^,^18^,^19^ So far, five (8.3%) deaths have been reported in this cohort, all of which were treatment-related and caused by neutropenic infections or hemorrhage. This mortality rate is significantly higher than that reported in developed countries, where it ranges from 2-4% in various trials.^20^,^21^ Many variables, including poor socioeconomic level, malnourishment, delayed presentation, and a lack of appropriate supportive services, exacerbate the toxic effects of chemotherapy in our circumstances.^22^ EFS was also evaluated with reference to t(1;19) positive and negative groups, and no statistically significant difference in survival was identified, which is consistent with recently reported studies.^14^,^17^-^19^ The most prevalent chromosome-1 aberration in the t(1;19) negative group was 1q duplication with hyperdiploidy. We observed no deaths or relapses in the 14 cases in this category, which is consistent with 100% EFS. This finding is in contrast to an Egyptian study which shows poor prognosis in this subgroup of patients.^13^ However, in our investigation, the shorter time of follow-up might be a limiting aspect.

The t(1;19) positive group was further classified as having balanced or unbalanced translocations. Though statistically insignificant (p-value = 0.155), there was a difference in survival, with 54% EFS for patients with balanced translocations and 81% for individuals with unbalanced translocations. In contrast, a prior study from Argentina found EFS of 88% and 78% for patients with balanced and unbalanced translocations, respectively.^12^

### Limitations of the study

This is a single-center, retrospectively analyzed data set with a short follow-up period. The lack of fluorescent in-situ hybridization (FISH) data for chromosomal abnormalities was the study’s main shortcoming. Furthermore, the results of minimal residual disease (MRD) were not used to determine the association with C1As.

## CONCLUSION

There were more unfavorable clinical features in t(1;19) patients, such as a higher median age, a higher white cell count, and more CNS positives. However, the EFS was comparable in both the t(1;19) positive and negative groups. Similarly, no negative association was found between 1q duplication and EFS. A difference in survival is noticed between patients with balanced and unbalanced t(1;19) aberrations. Although statistically insignificant, these results are still preliminary and require further large-scale prospective studies to gain more insight. Moreover, low EFS in the entire cohort is worrisome and warrants efforts to address the reasons for high treatment-related mortality and abandonment rates.

### Authors Contribution:

**NM:** Conceived, designed, drafted the manuscript and responsible and accountable for the accuracy and integrity of the work.

**NR:** Data collection and analysis.

**SJ:** Critical review and editing of manuscript.

**AE:** Critical review and final approval.

All authors have approved the final manuscript.
